# Deep Learning Model for Predicting Operative Mortality After Total Gastrectomy: Analysis of the Japanese National Clinical Database (NCD)

**DOI:** 10.1002/ags3.70067

**Published:** 2025-07-16

**Authors:** Ryosuke Fukuyo, Hiroyuki Yamamoto, Masanori Tokunaga, Yusuke Kinugasa, Yoshihiro Kakeji, Ken Shirabe

**Affiliations:** ^1^ Department of Gastrointestinal Surgery Institute of Science Tokyo Tokyo Japan; ^2^ Department of Healthcare Quality Assessment, Graduate School of Medicine The University of Tokyo Tokyo Japan; ^3^ Division of Gastrointestinal Surgery, Department of Surgery Kobe University Graduate School of Medicine Kobe Japan; ^4^ Department of General Surgical Science Gunma University Graduate School of Medicine Maebashi Gunma Japan; ^5^ The Japanese Society of Gastroenterological Surgery Tokyo Japan

**Keywords:** big data, deep learning, gastrectomy, gastric cancer, postoperative complications

## Abstract

**Background:**

Radical gastrectomy with lymph node dissection is the primary treatment for gastric cancer. However, the overall complication rate remains approximately 10%–20%, with a postoperative mortality rate of 2.3%. Therefore, preoperative stratification of patients based on their expected surgical risks is important. This study aimed to develop a deep learning prediction model using big data from the National Clinical Database (NCD) to predict operative mortality after total gastrectomy.

**Methods:**

Patients aged 18 years or older who underwent total gastrectomy for gastric cancer and were registered in the NCD between January 2018 and December 2019 were included. A total of 62 variables, including age, sex, past medical history, preoperative blood test results, and tumor characteristics, were used as covariates, with operative mortality as the outcome variable. Deep learning models were developed using Python, TensorFlow and Keras. Hyperparameters were adjusted using the k‐fold method with the training data. The model was evaluated using validation data.

**Results:**

Of the 14 980 eligible cases, 11 980 were used for training and 3000 for validation. The event rate was 1.2%. A four‐layer, 5217‐variable model was developed. The final C‐statistic was 0.79 (95% confidence intervals: 0.74–0.83) for the training data and 0.74 (95% confidence intervals: 0.62–0.85) for the validation data.

**Conclusion:**

We developed a deep learning model to predict operative mortality using big data from the NCD. To improve the accuracy, it is necessary to introduce new variables related to postoperative complications or factors that cannot be analyzed using conventional methods.

## Graphical abstract

Deep learning‐based prediction model for operative mortality using the National Clinical Database (NCD). The model achieved a C‐statistic of 0.74.

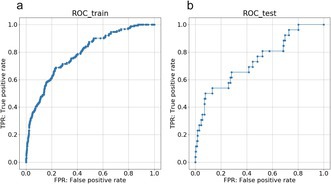

## 1 Introduction

Gastric cancer is one of the leading causes of cancer‐related deaths worldwide and is particularly important in Japan, where it is the third leading cause of cancer‐related deaths [1, 2]. The primary treatment for gastric cancer is radical gastrectomy with lymph node dissection. Recently, minimally invasive surgery has gained wider acceptance, with gradual improvements in safety. However, the overall complication rate remains approximately 10%–20% [3]. Additionally, total gastrectomy is associated with higher morbidity and mortality rates than distal gastrectomy [3].

Recent reports have suggested an association between postoperative complications and survival outcomes [4, 5, 6, 7, 8, 9, 10]. Some postoperative complications, such as anastomotic leakage or pancreas‐related complications, can be fatal and directly lead to operative mortality. Even when postoperative complications are successfully managed, affected patients are susceptible to disease recurrence [4, 11, 12]. Therefore, although curative surgery is essential for patients with gastric cancer, it is necessary to stratify patients by their expected surgical risks and offer individualized treatment to high‐risk patients, particularly those prone to operative mortality [4, 5, 8, 13].

The Japanese National Clinical Database (NCD) is a nationwide, web‐based database that includes 5679 Japanese institutions with 2.63 million surgeries annually. As of 2024, 28.48 million surgeries have been registered. The NCD risk calculator, developed using preoperative data, predicts operative mortality for each procedure and is widely utilized in Japan [14, 15, 16, 17]. By entering a patient's preoperative data into the NCD risk calculator web system, the expected mortality rate is calculated and displayed, enabling surgeons to make more informed decisions on the surgical procedure. For example, if the expected mortality rate is too high to ignore, the surgeon can omit prophylactic lymph node dissection or recommend non‐surgical treatments [18].

However, the accuracy of the current predictive model is still limited, and more accurate models are expected. Deep learning and machine learning techniques have been used to develop predictive models in various fields with remarkable results. With deep learning, it has been reported that the larger the dataset, the more accurate the predictive value tends to be. Because the NCD is one of the largest surgical databases in Japan, it is expected that a deep learning model would be well‐suited to the NCD dataset, making it possible to establish a more accurate predictive model.

Given the increasing proportion of patients with upper‐third gastric cancer and esophagogastric junction cancer, the number of patients undergoing total gastrectomy is expected to rise. This trend is concerning owing to the higher operative morbidity and mortality rates associated with total gastrectomy than distal gastrectomy [3, 13, 14, 15, 16, 17, 19, 20]. Deep learning and machine learning techniques have been used to develop predictive models in various fields with remarkable results [21, 22, 23]. Integrating these techniques into current models could enhance the accuracy of predicting early surgical outcomes, including operative mortality, leading to more personalized treatment options. This study aimed to develop a deep learning prediction model using big data from the NCD to predict operative mortality after total gastrectomy.

## 2 Materials and Methods

### 2.1 Patient Selection

**FIGURE 1:**
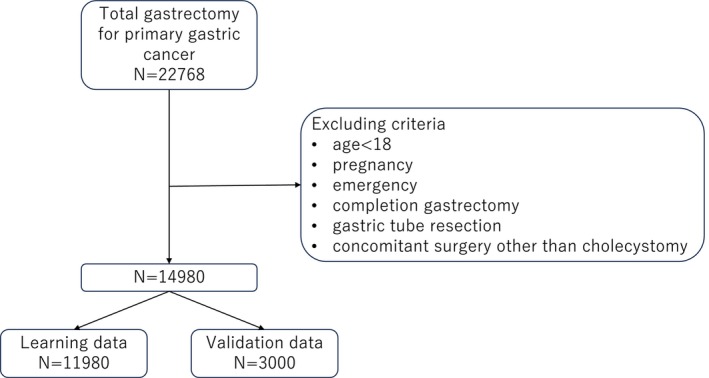
Study selection flowchart. Out of 22 768 cases of total gastrectomy, 14 980 cases are included after the exclusion criteria; 11 980 cases are used for model training, and the remaining 3000 for validation.

Patients aged 18 years or older who underwent total gastrectomy for gastric cancer between January 2018 and December 2019 were included in this study. Patients were excluded if they underwent concomitant resection of organs other than the gallbladder, had stage IV disease, underwent emergency surgery, or were pregnant. The inclusion and exclusion criteria of this study, along with the selection process, are shown in Figure 1.

### 2.2 Data Selection

The covariates used in this study were preoperative clinical and oncological factors. Clinical factors included height, weight, blood test results, history of chemotherapy, pre‐existing medical conditions such as diabetes and hypertension, and the American Society of Anesthesiologists physical status (ASA‐PS) classification. Oncological factors included tumor location and TNM classification. A list of variables and categorization criteria is presented in Table 1. The selection of variables was based on previous reports, but the number of variables was increased because it was believed that deep learning could handle more complex data. Since the preoperative risk classification is intended to be used for modifying the surgical procedure and consideration of the treatment plan, only information available at the start of surgery was employed. Therefore, information such as operative time, blood loss, details of the procedure, degree of lymph node dissection and presence or absence of postoperative complications was not employed. All numerical data were categorized and used with reference to previous reports [14, 15, 16, 17, 18].

**TABLE 1:** Variables and categorization.

Variable	Categories						
Patient characteristics							
Sex	Male	Female					
Age	18–60	61–70	71–80	81–90	91–119		
History of chemotherapy	No	Yes					
History of radiation	No	Yes					
Height	−140	141–160	161–180	181–200			
Weight	−30	31–50	51–70	71–90	91—		
Body mass index	−15	16–20	21–25	26–30	31—		
Diabetes	No	Yes (dietary treatment only)	Yes (internal treatment)	Yes (insulin)	Yes (no treatment)		
Brinkman Index	0	1–100	101–500	501–1000	1001—		
Alcohol	Never	Occasional drinking	Habitual				
Dyspnoea	No	On moderate exertion	Even at rest				
Activities of daily living	Independent	Partial support	Total support				
Respirator	No	Yes					
Chronic obstructive pulmonary disease	No	Yes					
Pneumonia on admission	No	Yes					
Encephalopathy	No	Slight	Sometimes in coma	Coma			
Ascites	No	Yes (no treatment)	Yes (controlled)	Yes (difficult to treat)			
Esophageal varices	No	Yes (no treatment)	Yes (controlled)	Yes (difficult to treat)			
Hypertension	No	Yes (no treatment)	Yes (with medication)				
Congestive heart failure	No	Yes					
History of myocardial infarction	No	Yes					
Angina	No	Yes					
Previous percutaneous coronary intervention	No	Yes					
History of cardiovascular surgery	No	Yes					
Previous surgery related to arterial occlusive	No	Yes					
Acute renal failure	No	Yes					
Dialysis	No	Yes					
Cerebrovascular disease	No	Yes (within 14 days)	Yes (15 days or earlier)				
Cancer with multiple metastases	No	Yes					
Open wound	No	Yes					
Long‐term steroid administration	No	Yes (withdrawal)	Yes				
Weight loss	No	Yes					
Bleeding risk factor	No (no anticoagulant therapy)	No (with anticoagulation)	Yes (no anticoagulant therapy)	Yes (with anticoagulation)			
Pre‐operative blood transfusion	No	Yes					
Preoperative sepsis	No	Sepsis	Septic shock				
Blood test							
White blood cell	< 3500	3500–9000	9001–12 000	12 001 <			
Hemoglobin	< 8.1	8.1–10	10.1–lower limit of normal	Normal range^a^	Upper limit of normal—		
Hematocrit	< 30.1	30.1—	Normal range^b^	Upper limit of normal—			
Platelet	< 80 001	80 001–149 999	150 000–350 000	350 001–400 000	400 000 <		
Albumin	< 2.1	2.1–3.0	3.1–3.9	4.0–5.0	5.0 <		
Total bilirubin	< 1.3	1.3–2	2.1–3	3.0 <			
AST (GOT)	< 36	36–150	150 <				
ALT (GPT)	< 36	36–150	150 <				
Alkaline phosphatase	< 110	110–340	341–600	600 <			
Blood urea nitrogen	< 8	8–20	21–100	100 <			
Creatinine	< 0.5	0.5–0.8	0.9–1.2	1.3–1.6	1.7–2.0	2.0 <	
Serum sodium	< 131	131–137	138–146	147–150	150 <		
Hemoglobin A1c	< 5.7	5.7–8.0	8.0 <				
C‐reactive protein	< 0.2	0.2–10.0	10.0 <				
Partial thromboplastin time (PT)	< 30	30–40	41–80	80 <			
ASA‐PS^c^	ASA1	ASA2	ASA3	ASA4	ASA5		
T Stage	T0	Tis	T1	T2	T3	T4a	T4b
N Stage	N0	N1	N2	N3			
Tumor epicenter	U	M	L				
Invasion of the esophagogastric junction	> 30 mm	21–30 mm	11–20 mm	1–10 mm	None		

^a^ Male: 13.5–17; Female: 11.5–15.

^b^ Male: 37%–48%; Female: 32%–42%.

^c^ American Society of Anesthesiologists Physical Status.

### 2.3 Outcomes

The objective variable was operative mortality, which included all deaths within 30 days after surgery and in‐hospital deaths, with a minimum postoperative follow‐up period of 90 days ensured by the database structure.

### 2.4 Data and Statistical Analysis

STATA17 (STATA Corp., TX, USA) was used for data handling.

Deep learning models were developed using Python (Van Rossum, 1995), TensorFlow and Keras. Datasets from 3000 patients were used as validation data, and the remaining were used as training data. The hyperparameters were adjusted using the k‐fold method with the training data. The Leaky Rectified Linear Unit (LReLU) function was used as the activation function, and a sigmoid function was used in the output layer to estimate the probability of event occurrence. The model was evaluated using validation data. The evaluation was conducted using the scikit‐learn package, which was used to generate the receiver operating characteristic (ROC) curve and calculate the C‐statistics. The Python environment and package versions used in the analysis are listed in Table A1 of Appendix 1.

## 3 Result

### 3.1 Patient Background

A total of 22 768 total gastrectomies for gastric cancer were registered, of which 14 980 were eligible for inclusion. The patient's background data are summarized in Table 2. Hypertension (43.9%) was the most common comorbidity, followed by diabetes mellitus (13.9%) and chronic obstructive pulmonary disease (5.6%). The proportion of patients with ≥ cT3 stage tumor and ≥ cN1 lymph node involvement was 72.4% and 51.0%, respectively. Preoperative chemotherapy was administered to 7.3% of patients. A total of 177 operative mortalities were observed (1.2%).

**TABLE 2:** Patients background.

Variables	*N*	%
Sex, male (%)	11 077	73.9
Age		
18–70	6544	43.7
71 <	8436	56.3
ASA‐PS		
1–3	14 940	99.7
4, 5	40	0.3
History of chemotherapy	1100	7.3
History of radiation	13	0.1
Past medical history		
Hypertension	6569	43.9
Diabetes	2088	13.9
COPD^a^	837	5.6
Angina	207	1.4
Acute renal failure	8	0.1
Dialysis	72	0.5
Long term steroid administration	161	1.1
Blood transfusion	409	2.7
Tumor		
Location		
U	9780	65.3
M	7806	52.1
L	2818	18.8
T stage		
T0	29	0.2
Tis	42	0.3
T1	4058	27.1
T2	2045	13.7
T3	4199	28
T4a	4199	28
T4b	408	2.7
N stage		
N0	7338	49
N1	2648	17.7
N2	2498	16.7
N3	2496	16.7

^a^ Chronic obstructive pulmonary disease.

### 3.2 Model Training

**FIGURE 2:**
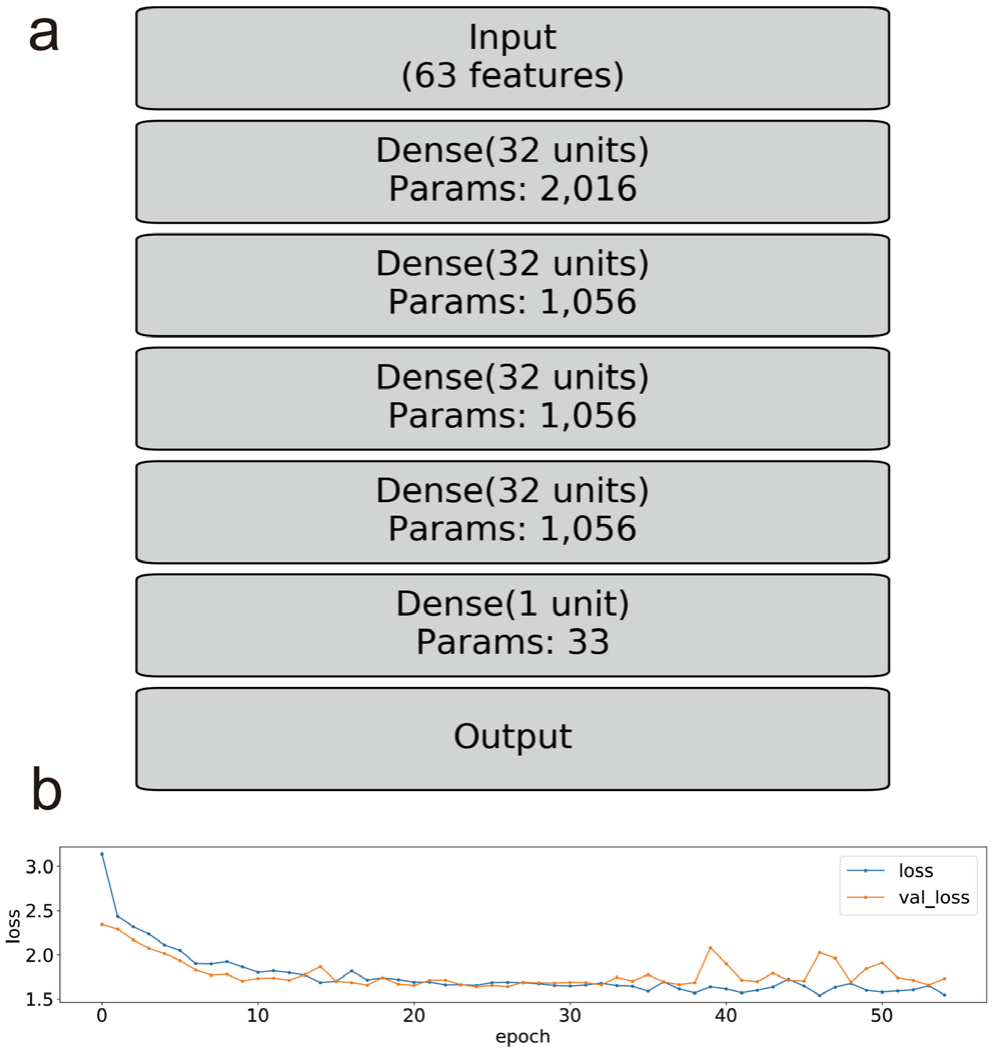
Loss function for the model in the teaching process. (a) Schema of the model. Each layer is connected by a Dense layer, and Rectified Linear Unit (ReLU) is used as the activation function. A total of 5217 variables are used. (b) Loss function during model training. Binary cross‐entropy is used for the loss function, and Adam is utilized as the optimization algorithm. The hyperparameters of the model are adjusted using the k‐fold cross‐validation method, and 5217 variables in four hidden layers are used. The L2 norm = 0.01 is used for regularization.

The hyperparameters of the model were adjusted using the k‐fold method. A four‐layer model incorporating 5217 variables was developed. Regularization was performed using an L2 norm of 0.01 to improve model generalizability, and 40% of the nodes were dropped during training. Binary cross‐entropy was used for the loss function, weighted by the inverse of the event probability, to address the class imbalance caused by the low event rate (~1%). The progress of the loss function during training is illustrated in Figure 2.

### 3.3 Model Evaluation

**FIGURE 3:**
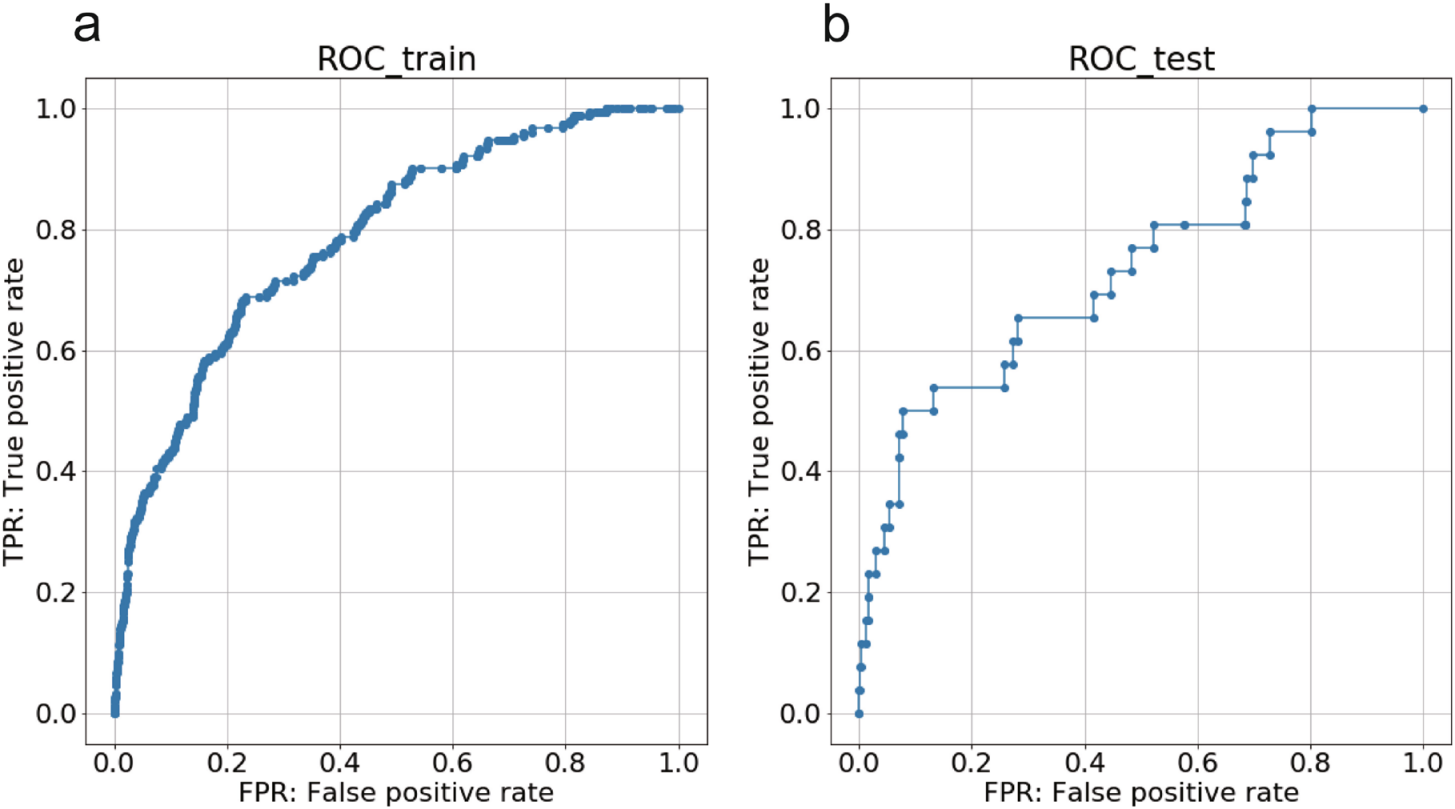
ROC curve of the predicting model. (a) ROC curve for the training data. C‐statistic is 0.79 (95% CI: 0.74–0.83). (b) ROC curve for the validation data. C‐statistic is 0.74 (95% CI: 0.62–0.85). CI, confidence interval; ROC, receiver operating characteristic.

**FIGURE 4:**
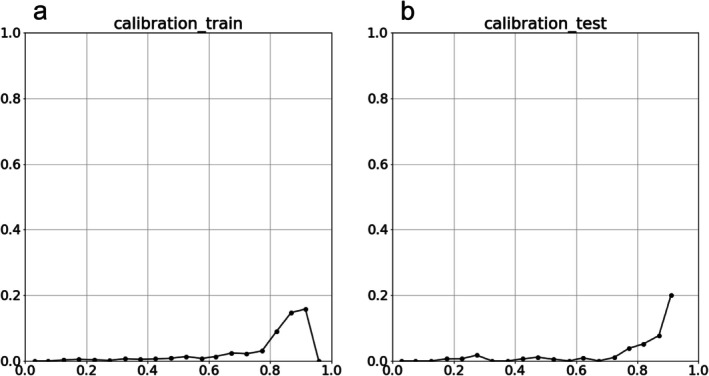
Calibration curves. (a) Calibration plot for training data. (b) Calibration plot for validation data.

The model was evaluated using a validation set of 3000 patients. The C‐statistic was 0.79 (95% confidence interval [CI]: 0.74–0.83) for the training data and 0.74 (95% CI: 0.62–0.85) for the validation data (Figure 3). Calibration plots for the model are shown in Figure 4.

## 4 Discussion

In this study, a deep learning‐based prediction model for operative mortality was developed using big data from the NCD. The model achieved a C‐statistic of approximately 0.74. This is the first attempt to use NCD data for training a deep learning model to predict operative mortality. Deep learning typically requires large datasets for effective training, and the NCD, which involves 5679 institutions in Japan and includes data from 28.48 million surgeries, provides an ideal database for such development.

Previous studies have used the NCD to create prediction models using traditional statistical methods. Watanabe et al. developed an operative mortality risk calculator for total gastrectomy, achieving a C‐statistic of approximately 0.811 [14]. Similarly, Kikuchi et al. developed a model to predict the risk of individual complications after total gastrectomy [15]. For distal gastrectomies, Kurita et al. [16] developed a mortality risk model that is currently available as a risk calculator on the NCD website. These models are widely used in Japan for preoperative informed consent and surgical decision‐making [18]. While these models have demonstrated significant utility in terms of predicting postoperative mortality, it has been anticipated that deep learning could enhance prediction accuracy for surgical morbidity and mortality. In the present study, we set postoperative mortality as an objective variable to simply compare predictive value with previous reports.

In the present study, the prediction accuracy of the deep learning model (C‐statistic: 0.74) did not surpass that of Watanabe et al.'s model [14]. We conducted this study in the hope that an increase in prediction accuracy would allow us to make changes in treatment, such as offering non‐surgical treatment to high‐risk patients or considering less invasive surgery. In addition, surgeons could consider transferring patients to a more experienced surgeon at a high‐volume center if the predicted mortality is extremely high. Therefore, we did not include surgeons' experience and hospital volume, which would affect the mortality rates, in the model. Nevertheless, the present model failed to outperform the previous model in terms of predicting postoperative mortality. Several factors may explain this outcome. First, since 2018, robot‐assisted surgery has been included in the NCD database and covered by the national insurance system. Consequently, the proportion of robotic gastrectomies in the present study was higher than in earlier studies. The surgeon's skill, which was not included as a covariate in either the present or previous models, likely influences early surgical outcomes, especially for newly developed procedures. Accordingly, the accuracy of predicting operative mortality may not be comparable with that observed in previous studies. Second, given that the patients in the present study underwent surgery after the widespread adoption of the NCD risk calculator, surgeons might have used the results of the risk calculator when considering the treatment strategy. As a result, surgeons might have offered non‐surgical treatments to patients with a predicted high operative mortality. Therefore, it may not be appropriate to directly compare the predictive accuracy of the present study's model with that of previous studies.

Deep learning has been reported to be useful in image recognition tasks, such as in computed tomography (CT) analysis and skin cancer diagnosis [21, 22]. Beyond the medical field, it has also been used to analyze complex systems, including natural language processing and image recognition [24]. Given the size and complexity of the NCD database, it was expected that the current study's deep learning model would outperform previous models in predicting operative mortality. However, its accuracy was not superior to previous models. One potential reason is the nature of the dataset. This study utilized 62 categorical variables, which, while relatively large for conventional forecasting models, may be insufficiently complex for deep learning applications. Previous studies have indicated that traditional logistic regression models can outperform deep learning for simpler datasets, such as tabular data [25]. In the present study, we used NCD, which is a relatively large data set, and conducted the analysis in the belief that even tabular data, which is generally considered a weak point, would yield sufficiently useful results. However, it was found that an increase in accuracy could not be expected with only tens of thousands of data points with our deep learning model. Further improvement in accuracy may require the integration of new data related to mortality and complications or the inclusion of complex data, such as medical images or surgical videos, which have not been previously analyzed. Future analyses integrating these data types could significantly improve the predictive accuracy of the model. We hope that postoperative complications and long‐term prognosis can also be predicted, and that safer and more reliable management will become possible for all patients with gastric cancer in the future.

This study has several limitations. First, the NCD is a domestic Japanese database predominantly comprising Japanese patients. Gastric cancer characteristics, patient demographics, and surgical practices vary widely across countries and regions, limiting the generalizability of this model in international settings. Second, deep learning uses complex functions to generate predictions from the input data, making it difficult to examine how individual variables are used to make predictions. Therefore, surgeons often hesitate to rely on ‘black‐box’ models for critical surgical decisions, even if the predictive value outperforms previous logistic models.

## 5 Conclusion

Using big data from the NCD, we developed a deep learning‐based predictive model for operative mortality after total gastrectomy. Although the model's predictive accuracy did not meet expectations. Although a nationwide database has become available in many countries, most databases have only tabular data, and therefore, including non‐text data should be considered when developing a risk calculation model with AI. We hope that future advancements in deep learning models will improve patient risk stratification, leading to appropriate treatment selection.

## Author Contributions

**Ryosuke Fukuyo:** conceptualization, methodology, software, investigation, validation, visualization, writing – original draft, writing – review and editing, formal analysis. **Hiroyuki Yamamoto:** data curation, conceptualization, validation, investigation, methodology, software, formal analysis, visualization, resources, writing – review and editing, project administration. **Masanori Tokunaga:** writing – review and editing, conceptualization, methodology, investigation, validation, project administration. **Yusuke Kinugasa:** conceptualization, methodology, writing – review and editing, supervision, project administration. **Yoshihiro Kakeji:** conceptualization, methodology, writing – review and editing, supervision, resources, project administration, funding acquisition. **Ken Shirabe:** conceptualization, supervision, writing – review and editing, funding acquisition, resources, project administration.

## Ethics Statement

The protocol for this research project has been approved by a suitably constituted Ethics Committee of the institution and it conforms to the provisions of the Declaration of Helsinki. This study was approved by the Institutional Review Board at our institute (approval number: M2020‐264). Since this was a retrospective data management study, individual written consent was not obtained, and an opt‐out notice was posted as a substitute for a consent form.

## Conflicts of Interest

Hiroyuki Yamamoto is affiliated with the Department of Healthcare Quality Assessment at the University of Tokyo. This department is a social collaboration department supported by grants from the National Clinical Database, Intuitive Surgical Sarl, Johnson & Johnson K.K. and Nipro Co. Yusuke Kinugasa is an associate editor member of Annals of Gastroenterological Surgery. Yoshihiro Kakeji and Ken Shirabe are editorial board members of Annals of Gastroenterological Surgery. The other authors declare no conflicts of interest.

## Appendix 1

**TABLE A1:** Python environments and package versions.

Package	Version
keras	2.3.1
numpy	1.17.2
tensorflow_gpu	1.15.0
scikit‐learn	0.22
matplotlib	3.1.1
pandas	0.25.2
